# Extraction of bioactive compounds from *Psidium guajava* and their application in dentistry

**DOI:** 10.1186/s13568-019-0935-x

**Published:** 2019-12-28

**Authors:** Shaik Shaheena, Anjani Devi Chintagunta, Vijaya Ramu Dirisala, N. S. Sampath Kumar

**Affiliations:** 1 Department of Biotechnology, Vignan’s Foundation for Science, Technology and Research, Guntur, Andhra Pradesh 522213 India; 2 Department of Molecular Biosciences, Faculty of Life Sciences, Kyoto Sangyo University, Kyoto, 603-8555 Japan; 3 Advanced Technology Development Centre, Indian Institute of Technology, Kharagpur, West Bengal 721302 India

**Keywords:** Antimicrobial activity, Cleansing ability, Formulation, Guava, Herbal toothpaste

## Abstract

Guava is considered as poor man’s apple rich in phytochemicals with medicinal value and hence it is highly consumed. Gas chromatography–mass spectroscopy (GC–MS) analysis of guava leaf extract revealed the presence of various bioactive compounds with antimicrobial, antioxidant, anticancer, and antitumor properties. Hence, it is used in tooth paste formulations along with other ingredients such as *Acacia arabica* gum powder, stevia herb powder, sea salt, extra virgin coconut oil, peppermint oil in the present study. Three formulations F1, F2 and F3 have been made by varying the concentration of these ingredients and the prepared formulations were studied for their antimicrobial activity and physico-chemical parameters such as pH, abrasiveness, foaming activity, spreading and cleaning ability. Among these, F3 showed significant antioxidant and antimicrobial properties, minimal cytotoxicity, maximum spreadability and very high cleaning ability. This study surmises that the herbal toothpaste formulation is greener, rich in medicinal values and imparts oral hygiene.
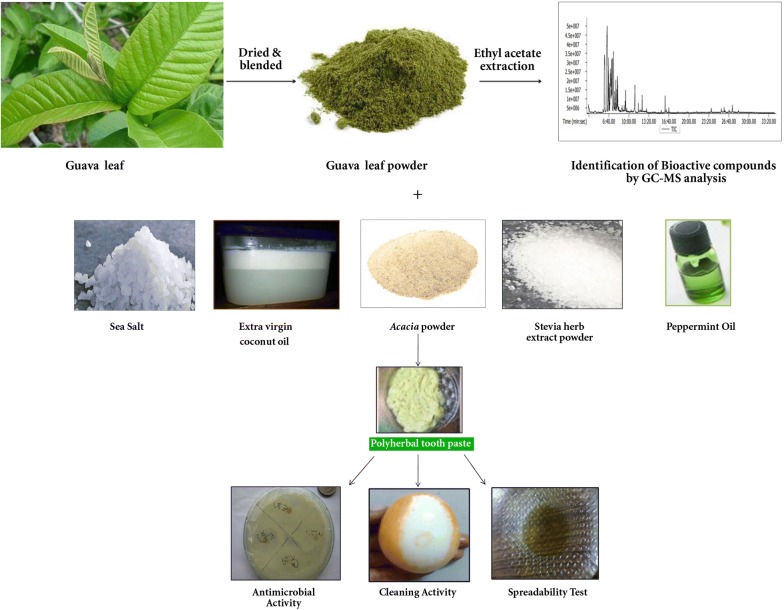

## Introduction

*Psidium guajava* (Guava) is an evergreen tree that belongs to the family *Myrtaceae*, grows in tropical and subtropical regions but preferably in dry climates. It is originated from Mexico or Central America and due to its health benefits it is grown abundantly in various countries that include Brazil, Bangladesh, China, Indonesia, India, Nigeria, Mexico, Pakistan, Thailand and Philippines (Uzzaman et al. 2018). It is commercially cultivated in almost all the states of India. In the year 2016–2017, the total estimated area under guava cultivation was 2,61,700 hectares (ha) with the production of 36,48,200 million tons (MT) (Horticultural Statistics at a Glance 2017). Guava is considered as multipurpose medicinal tree similar to *Mangifera indica* and *Azadirachta indica* because of myriad medicinal values from various parts viz., leaf, roots, bark and fruit (Sravani et al. 2015; Naidu et al. 2016; Raju et al. 2019). The leaf extract of guava has pharmacological activity (Uzzaman et al. 2018) due to the presence of bioactive compounds that treat dysentery, diarrhoea, flatulence, gastric problems and regulate blood glucose levels. The guava leaves contain essential oils rich in cineol, triterpenes, tannins, eugenol, kaempferol and other compounds such as flavonoids, malic acid, gallic acid, chlorophyll and mineral salts (Kumar et al. 2019). To impart the beneficial aspects of the guava leaf extract to the daily used products, an attempt was made to formulate a tooth paste with guava leaf extract as a major ingredient in the present work.

Inconsistent eating habits and high sugar consumption encourage the growth of bacteria leading to various oral diseases. Approximately, 600 bacterial species are estimated to exist in the human oral microbiome (Bora et al. 2014) among which, some are involved in protecting the mouth while the rest are responsible for causing oral diseases. The bacteria ferment sugars and starch into acid which dissolves the minerals in the tooth enamel and leads to decalcification and formation of tooth decay/cavities. In order to maintain the oral health, the bacterial growth should be prevented by including the bioactive compounds with antimicrobial properties in toothpaste formulation (Vijaya et al. 2017).

In general, commercially available toothpastes contain ingredients to enhance properties like antimicrobial, antioxidants, aesthetic appeal, surfactants, thickening agent to change rheological properties, preservatives and binders to provide consistency and stability for formulations (Das et al. 2013). Unfortunately they will have fluorides, strong abrasives, sodium lauryl sulphate, colouring dyes and other agents like triclosan that have negative impact in maintaining healthy gums and teeth. Besides, large number of chemicals can cause damage to the enamel and gums. So, in this paper we have formulated a herbal toothpaste with guava leaf extract, *Acacia arabica* powder, sea salt, stevia herb extract powder which were scientifically proven to be harmless, carcinogen free natural source with high therapeutic value.

## Materials and methods

### Sample processing

Fresh green leaves of guava (*Psidium guajava*) tree were collected from the premises of Vignan’s Foundation for Science, Technology and Research, Vadlamudi, Guntur, Andhra Pradesh. The leaves were gently rinsed with water, sundried to remove the moisture and powdered using a blender. The powder was then passed through aluminium sieve of (1 mm) to get uniform particle size. Guava leaf powder was stored in an air tight container for further studies.

### Guava leaf extraction procedure

The guava leaf powder (25 g) was suspended in ethyl acetate (100 mL) and stirred for 24 h under sterile conditions (Seo et al. 2014). The extract was filtered using Whatman no. 1 filter paper and the filtrate was used for identification of various phytochemicals/bioactive compounds based upon the retention time and mass spectra of the library retrieved from National Institute of Standards and Technology (NIST).

### Gas chromatography–mass spectroscopy (GC–MS) method

GC–MS analysis was carried out in Agilent Technologies, Gas Chromatograph 7890 and Mass Spectrometer 5975. DB-5HT nonpolar capillary column (30 m × 0.25 mm × 0.1 μm) manufactured from (5%-phenyl)-methylpolysiloxane was used for the identification of phytochemicals. Helium gas was used as carrier gas with a consistent flow rate of 1.2 mL/min, sample injection volume was 0.5 μL and the ion-source temperature was 230 °C. The oven temperature was programmed from 80 °C (isothermal for 1 min), with an increase of 10 °C/min, to 200 °C, then 5 °C/min to 300 °C. Mass spectra were taken at 70 eV; a scan interval of 10 spectra/s and fragments from 50 to 800 Da. The relative percentage of each component can be calculated by comparing its average peak area to the total areas. The spectrum of the unknown component can be compared with the spectrum of the known components stored in the NIST library.

### Ingredients for paste preparation

Sea salt, *Acacia arabica* gum powder, stevia herb extract powder (procured), extra virgin coconut oil and peppermint oil were used as ingredients for formulation of toothpaste.

### Preparation of ingredients

#### Extra virgin coconut oil

Fresh coconuts were collected and grated. Small quantity of water was added to the pressed and mashed coconut and left for 30 min for extraction of coconut milk. Filtration was carried out through the cheese cloth and the filtrate was left overnight at 25 °C for the separation of coconut cream (top layer) and extra virgin oil (bottom layer). Oil was separated and stored in the refrigerator.

#### Peppermint oil

Fresh leaves of menthe were collected, pressed slightly to release the oil and blended with almond oil for 24 h. Residual leaves were removed and fresh leaves were added at a regular interval of 24 h and the process was continued for a week to obtain peppermint essential oil. The oil was stored in an air tight container away from light.

#### *Acacia arabica* gum powder

*Acacia* gum was purchased, air dried and ground to get fine powder using mechanical mixer and stored in an air tight container.

### Formulation of toothpaste

Three formulations of tooth paste were prepared by varying the concentrations (%) of ingredients viz, guava leaf powder, *Acacia arabica* gum powder, sea salt, stevia herb extract powder and pepper mint oil (Table 1). *Acacia arabica* gum powder was mixed with small quantity of distilled water using a dropper to make a smooth paste. Subsequently sea salt, guava leaf powder and stevia herb extract powder were added and mixed well to make the paste uniform. Consequently, extra virgin coconut oil and pepper mint oil were added and mixed well until the toothpaste attains the desired consistency. F1, F2 and F3 pastes were packed and stored in plastic jars.

**Table 1:** Composition ratio of ingredients used in different tooth paste formulations

Ingredients	Property	F1 (%)	F2 (%)	F3 (%)
*Acacia arabica* gum powder	Binder	15	12.5	10
Sea salt	Abrasiveness	10	7.5	5
Guava leaf powder	Anti-inflammatory, anti-mutagenic, anti-microbial, abrasiveness, preservation	10	15	20
Stevia herb extract powder	Sweeter	15	12.5	10
Coconut oil	Reduces plaque built up, prevent tooth decay, fight gum diseases, essential oil, humectant activity	5	5	5
Pepper mint oil	Flavouring agent, reduces plaque built up, improves salivary buffer capacity, decreases salivary *S. mutant* count	5	7.5	10
Distilled water	Solvent	40	40	40

### Physico-chemical evaluation of toothpaste

To assess the tooth paste formulations, physicochemical properties of the paste were estimated. All the assays were conducted in triplicates and data was represented as mean ± standard deviation. The statistical analysis was performed using SPSS 10.0 software. Significant differences were determined with 95% confidence interval (P < 0.05).

### Determination of pH

Toothpaste solution (2%, w/v) was prepared and the pH was determined at room temperature using a calibrated pH meter.

### Determination of abrasiveness

A pea-sized dab of toothpaste was placed on a clean plastic slide and few drops of distilled water were added to it. Then the toothpaste sample was rubbed in back and forth motion for 25 times within a distance of 1 cm using a fresh cotton swab. Then the slide was carefully rinsed, dried with tissue paper and examined under a microscope to determine the number of scratches on the surface of the slide. The degree of scratches was rated from 0 (no scratches) to 5 (a high degree of scratches).

### Determination of foaming activity

Toothpaste (1 g) was mixed with distilled water (15 mL) in a measuring cylinder, shaken vigorously for 1 min, placed on the table to measure the height of the foam above the water level (Das et al. 2013). The foaming ability of the toothpaste was determined using the following equation.

1 $$Foaming\;ability(\% ) = \frac{{Height\;of\;the\;foam\;above\;water}}{{Total\;height}} \times 100$$

### Spreading ability test

Toothpaste (1 g) was laid at the centre of a glass slide and covered with another glass slide. A known weight (1 kg) was placed carefully on these slides for 10 min to allow the paste to spread and then the diameter of the paste was measured (Mangilal and Ravikumar 2016).

### Cleaning ability test

The composition of the eggshell is similar to the teeth enamel with calcium as the major compound. For this reason boiled eggs were used for testing the cleaning ability of the formulated tooth pastes as reported by Das et al. (2013) with necessary modifications. In boiling water, vinegar and few drops of food colour (red) were added. After cooling, the boiled eggs were immersed and allowed to stain for 5 min at 25 °C. The eggs were removed from the food colouring solution and placed on a paper towel to remove access water. Then the eggshell was washed with a wet tooth brush without losing the colour of stain followed by washing with known quantity of toothpaste. As per the requirement, 5–10 brush strokes with F1, F2 and F3 toothpastes on eggshell were given for colour removal. Same kind of pressure and motion were used in brushing procedure for all the three formulated toothpastes. The cleaning ability of the three toothpaste formulations were observed and the results were interpreted as ‘+++’ very high cleaning ability, ‘++’ high cleaning ability, ‘+’ moderate cleaning ability, ‘−’ bad cleaning ability.

### Determination of antimicrobial activity

#### Antimicrobial assay

Antibacterial activity of the three toothpaste formulations was evaluated against five strains of microorganisms: *Bacillus subtilis* (MTCC 1305), *Proteus vulgaris* (MTCC 744), *Staphylococcus aureus* (MTCC 9760), *Streptococcus mutants* (MTCC 890) and *Streptococcus oralis* (MTCC 2696) using the well diffusion method. The inoculum of these bacterial strains was prepared in LB medium and incubated at 37 °C for 24 h. Nutrient agar plates (LB) were inoculated with each test microorganism (1 mL of the broth cultures) and dried for 1 h. Ampicillin (50 mg/mL) was used as a positive control. Solutions (2%w/v) of the three tooth paste formulations (F1, F2 and F3) were prepared and 60 μL of each formulation was poured in the designated well. The plates were then kept for 2 h in the refrigerator for diffusion of samples and then incubated at 37 °C for 24 h.

#### Calculation of zone of inhibition

After incubation, the zone of inhibition appears as a clear and circular halo around the wells. The diameter of the circular halo was measured both vertically and horizontally and their average was considered (cm).

#### In vitro cytotoxicity test

Vero cells were procured from National Centre for Cell Sciences, Pune, India and maintained in Dulbecco’s Modified Eagle’s Medium (DMEM) supplemented with Foetal bovine serum (FBS, 10%), l-glutamine (2 mM), penicillin G sodium (100 U/mL) and streptomycin sulphate (100 mg/mL). Cells were seeded (1 × 10^4^ cells/mL) in aforementioned media in 96-well plate and incubated at 37 °C in 5% CO_2_. After reaching confluence, cytotoxicity of the cells was tested with various concentrations of formulated tooth paste. Medium was discarded after 24 h of incubation and the adherent cells were washed with phosphate buffer saline (PBS) and 30 µL of 3-(4,5-dimethyl-2-thiazolyl)-2,5-diphenyl-tetrazolium bromide (MTT; 10 mg/mL in PBS) and incubated for 6 h. Dimethyl sulfoxide (DMSO, 70 µL) was added to solubilise the formazan crystals produced by viable cells. The absorbance was measured at 540 nm using UV–Vis spectrophotometer (Shimadzu, Japan; Model: UV-1800). Cell viability (%) in the presence of tooth paste was measured and expressed as follows:

2 $$\% \;{Proliferation} = \left[ {OD_{{sample}} - OD_{{control}} } \right] \times \frac{{100}}{{OD_{{control}} }}$$

#### Stability studies

In order to study the stability of the formulated toothpaste, its physico-chemical properties were studied at a regular interval of 4 months for a period of 12 months.

## Results

### Extraction of guava leaf extract and identification of bioactive compounds

**Fig. 1:**
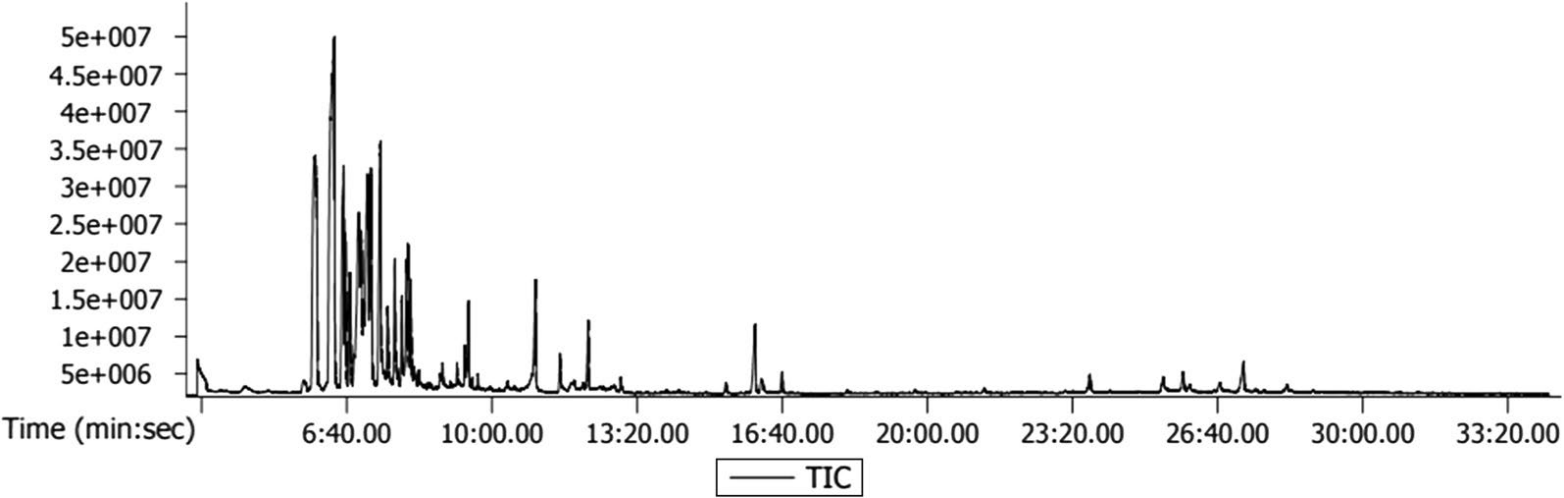
GC–MS chromatogram of bioactive compounds in guava leaf extract

In the present study, the extraction of bioactive compounds from the guava leaf was carried out using ethyl acetate. The ethyl acetate extract was subjected to GC–MS analysis which has manifested the presence of sesquiterpenes and fatty acids predominantly (Fig. 1, Table 2). Caryophyllene, α-copaene, *cis*-muurola-3,5-diene, humulene, cyclosativene, bicyclo[5.3.0]decane, 2-methylene-5-(1-methylvinyl)-8-methyl, 1*H*-benzocycloheptene, 2,4a,5,6,7,8,9,9a-octahydro-3,5,5-trimethyl-9-methylene-, (4aS-*cis*), 1H-cyclopropa[a]naphthalene, 1a,2,3,5,6,7,7a,7b-octahydro-1,1,7,7a-tetramethyl-, [1aR-(1aà,7à,7aà,7bà)], naphthalene, 1,2,3,5,6,8a-hexahydro-4,7-dimethyl-1-(1-methylethyl)-, (1S-*cis*), α-cadinol, α-bisabolol etc. are some of the sesquiterpenes identified in the guava leaf extract. These compounds are well known for their antimicrobial, anti-inflammatory, antioxidant, antiproliferative, anticancer, antitumors and anaesthetic properties (Zahin et al. 2017).

**Table 2:** Identification of phytochemicals in guava leaf extract by GC–MS

S. no.	Identified compounds	RT (min)	Formula	Area	Exact mass	Activity	Nature
1	Caryophyllene	6:22.5	C15H24	1568657760	204.188	Anaesthetic and anti-inflammatory activities. Gives fragrance and act as metabolite	Bicyclic sesquiterpene
2	α-Copaene	5:55.80	C15H24	1210708553	204.1878	Antiproliferative and antioxidant properties	sesquiterpene
3	*Cis*-muurola-3,5-diene	6:34.10	C15H24	369140884	204.1878	Flavoring agent	Carbobicyclic sesquiterpene
4	Humulene	6:35.80	C15H24	1031583140	204.1878	Flavoring agent, anti-inflammatory and analgesic agent	Monocyclic sesquiterpene
5	Cyclosativene	6:44.60	C15H24	370318123	204.188	Antiproliferative, genotoxic and oxidant activities	Tetracyclic sesquiterpene
6	Bicyclo[5.3.0]decane, 2-methylene-5-(1-methylvinyl)-8-methyl-	6:50.30	C15H24	27515480	204.188	Antibacterial, anticancer and antitumor activities	Present in sesquiterpenes and diterpenes
7	*cis*-à-Bisabolene	6:54.70	C15H24	240237972	204.1878	Intermediate in the biosynthesis of hernandulcin which is a natural sweetner	Sesquiterpene
8	1*H*-Benzocycloheptene, 2,4a,5,6,7,8,9,9a-octahydro-3,5,5-trimethyl-9-methylene-, (4aS-*cis*)-	7:00.20	C15H24	413675493	204.1878	Antioxidant activity	Diterpenoid
9	1*H*-Cyclopropa[a]naphthalene, 1a,2,3,5,6,7,7a,7b-octahydro-1,1,7,7a-tetramethyl-, [1aR-(1aà,7à,7aà,7bà)]	7:08.90	C15H24	649824928	204.1878	Antibacterial activity	Sesquiterpene
10	Naphthalene, 1,2,3,5,6,8a-hexahydro-4,7-dimethyl-1-(1-methylethyl)-, (1S-*cis*)	7:09.20	C15H24	279565355	204.1878	Plant metabolite	Sesquiterpene and a carbobicyclic compound
11	Benzene, (1,3,3-trimethylnonyl)-	7:12.80	C18H30	231301272	246.2348	Flavouring agent	Aromatic hydrocarbon
12	Cadala-1(10),3,8-triene	7:24.90	C15H22	291832784	202.1722	Anti-inflammatory and antibacterial activities	Sesquiterpene
13	1,6,10-Dodecatrien-3-ol, 3,7,11-trimethyl-, (E)-	7:26.90	C15H26O	427329786	222.1984	Flavoring and fragrance agents	Sesquiterpene alcohol
14	Spathulenol	7:48.10	C15H24O	48103606	220.1827	Plant metabolite, an anaesthetic and a vasodilator agent	Tricyclic sesquiterpenoid
15	Cubenol	7:46.60	C15H26O	339279382	222.1984	Flavor and fragrance agent	Sesquiterpene
16	6S-2,3,8,8-Tetramethyltricyclo[5.2.2.0(1,6)]undec-2-ene	7:57.40	C15H24	9681042	204.1878	Fragrance agent	Sesquiterpenoid
17	2-Hydroxy-2,4,4-trimethyl-3-(3-methylbuta-1,3-dienyl)cyclohexanone	8:02.80	C14H22O2	324578226	222.162	Antimicrobial activity	Cyclic ketone
18	Torreyol	8:04.40	C15H26O	275623562	222.1984	Antibacterial activity	Bicyclic tertiary alcohol
19	α-Cadinol	8:08.20	C15H26O	197840577	222.1984	Anti-fungal activity	Sesquiterpene alcohol
20	5,6,6-Trimethyl-5-(3-oxobut-1-enyl)-1-oxaspiro[2.5]octan-4-one	8:09.20	C14H20O3	31434205	236.1412	Anti-inflammatory, anti-tumor, anti-septic and anti-microbial activities	Spiro compound
21	α-Bisabolol	8:13.30	C15H26O	38007475	222.1984	Antiulcer, antiphlogistic effects, antimicrobial, antioxidant and anti-inflammatory activities	Monocyclic sesquiterpene alcohol
22	Tetradecanoic acid, trimethylsilyl ester	9:28.10	C17H36O2Si	218587280	300.2485	Fragrance ingredient	Fatty acid
23	10-Undecynoic acid, trimethylsilyl ester	9:28.30	C14H26O2Si	224076078	254.1702	Fragrance ingredient	Fatty acid
24	Hexadecanoic acid, trimethylsilyl ester	11:00.30	C19H40O2Si	470791453	328.2798	Plant metabolite	Hexadecanoate ester
25	Isoamyllaurate	24:54.3	C17H34O2	4387.8	270.2559	Fragrance ingredient	Fatty acid ester

### Evaluation of physical–chemical properties of tooth paste formulations

Various ingredients involved in the tooth paste formulation includes guava leaf powder, *Acacia arabica* gum powder, sea salt, stevia herb extract powder and pepper mint oil. Three formulations of herbal toothpaste (F1, F2 and F3) were prepared by varying the concentrations of these ingredients and their properties were studied to identify the best formulation (Table 1). The physical and chemical characteristics of different toothpaste formulations showed significant variations (Table 3). pH of all the toothpaste formulations was in the alkaline range of 8–11 in which F1 showed highest pH of 11.8 ± 0.01 and remaining two were in proximity to pH 9. Similar patterns were observed for abrasiveness and foaming ability tests also. Rubbing F1 against the glass slides, created more scratches than F2 and F3. Regarding the foaming ability, the F2 toothpaste showed lower foaming ability (15.1%) and F1 exhibited the highest value of 16.6%. Even though all the three formulations have shown less foaming properties, they showed very good spreading ability. F3 formulation has showed highest spreading area (8.1 ± 0.03 cm) followed by F2 and F1 with 7.8 ± 0.2 cm and 6.0 ± 0.5 cm respectively. Based on the colour change appeared on the pigmented eggs, F3 has shown better ability of cleaning stains (+++) and comparatively remaining two formulations (++) has shown less change in colour.

**Table 3:** Physico-chemical properties of tooth paste formulations

Formulations	pH	Abrasiveness (rating)	Foaming ability (%)	Spreadability (cm)	Cleaning ability
F1	11.8 ± 0.01	4	16.6 ± 0.3	6.0 ± 0.50	++
F2	09.6 ± 0.02	2	15.1 ± 0.1	7.8 ± 0.20	++
F3	08.1 ± 0.01	2	16.0 ± 0.4	8.1 ± 0.03	+++

### Antibacterial activity

In vitro antibacterial activity of the formulated toothpaste (F1, F2 and F3) were evaluated against *Bacillus subtilis*, *Proteus vulgaris*, *Staphylococcus aureus*, *Streptococcus mutants* and *Streptococcus oralis* strains as shown in Table 4. F3 formulation was very effective against the tested bacteria followed by F2 and least activity was shown for F1. As tabulated, the highest inhibition zone was observed against *Proteus vulgaris* (1.1 cm) and *Bacillus subtilis* (0.8 cm) and the lowest zone of inhibition was observed against *Staphylococcus aureus* (0.5 cm) for F3 formulated paste.

**Table 4:** Anti-microbial activity of tooth paste formulations

Formulations	*Bacillus subtilis* (cm)	*Proteus vulgaris* (cm)	*Staphylococcus aureus* (cm)	*Streptococcus mutans (cm)*	*Streptococcus oralis* (cm)
F1	0.3 ± 0.001	0.2 ± 0.002	0.4 ± 0.001	0.39 ± 0.02	0.4 ± 0.003
F2	0.6 ± 0.003	0.8 ± 0.003	0.7 ± 0.004	0.41 ± 0.005	0.3 ± 0.001
F3	0.8 ± 0.001	1.1 ± 0.006	0.5 ± 0.001	0.9 ± 0.006	0.6 ± 0.002

### In vitro cell viability assay

**Fig. 2:**
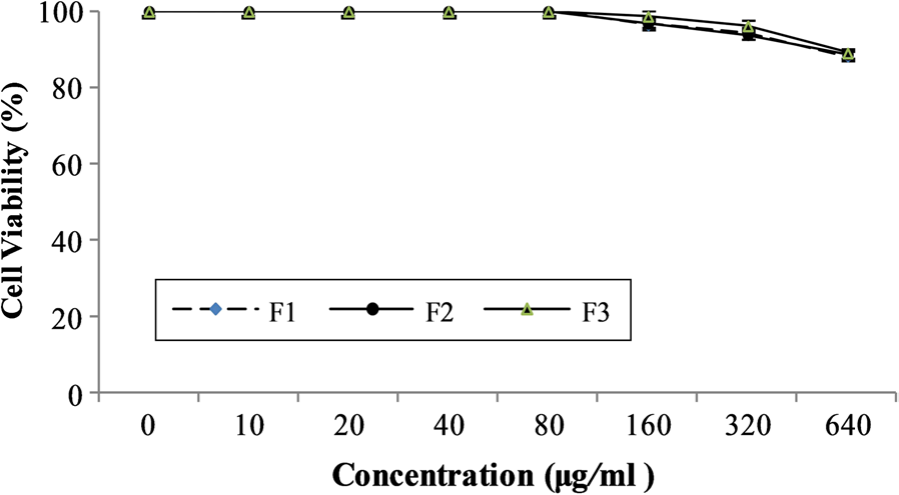
Cytotoxicity of formulated toothpaste against Vero cells

Cytotoxicity of formulated toothpastes at 0, 10, 20, 40, 80, 160, 320 and 640 μg/mL concentration for Vero cells was determined using MTT assay. Results have confirmed that tested toothpastes have no significant effect on the reduction of Vero cell viability (*P *< 0.05) up to the concentration of 320 μg/mL (Fig. 2). But, the cell viability decreased slightly at 640 μg/mL concentration of formulated toothpastes.

### Stability test

Upon comparing the physical, chemical and biological activities of all the three formulations, F3 was found to be more suitable and selected for stability test. The test was conducted for a period of 12 months at an interval of 4 months to observe changes in F3 during its storage period. As shown in Table 5, no significant change was observed in the pH, foaming ability, spreadability and cleaning ability of F3 inferring that it is best opted formulation for human application. Table 6 depicts the organoleptic evaluation of toothpaste formulation F3. Pale green colour and better taste of the paste is mostly imparted by the guava leaf extract.

**Table 5:** Stability test for tooth paste formulation (F3)

Duration	pH	Foaming ability (%)	Spreadability (cm)	Cleaning ability
4th month	8.0 ± 0.01	16.0 ± 0.2	8.1 ± 0.50	++
8th month	7.9 ± 0.02	16.0 ± 0.1	7.9 ± 0.20	++
12th month	7.9 ± 0.01	16.0 ± 0.1	7.9 ± 0.03	++

**Table 6:** Organoleptic analysis of tooth paste formulation (F3)

Organoleptic evaluation	F3
Colour	Pale green
Taste	Slightly bitter
Odour	Pleasant
Texture	Partially smooth

## Discussion

Guava leaf is rich in bioactive compounds and in order to utilize these compounds in the preparation of value added products, these are extracted using ethyl acetate. Seo et al. (2014) reported the extraction of essential compounds of guava using water, ethanol and methanol, and found highest content of phenolic compounds in water extract. The ethyl acetate extract was found to be rich in sesquiterpenes and fatty acids. Sesquiterpenes such as cubenol, 6S-2,3,8,8-tetramethyltricyclo[5.2.2.0(1,6)]undec-2-ene, benzene, (1,3,3-trimethylnonyl) and 1,6,10-dodecatrien-3-ol, 3,7,11-trimethyl-, (E) act as flavouring and fragrance agents. Apart from these, *cis*-à-bisabolene, an intermediate in the biosynthesis of hernandulcin which is a natural sweetener was also identified in the extract (Christianson 2017). Another major compound identified in the extract was fatty acid esters which are found to be fragrance ingredients. Thus, the GC–MS analysis of guava leaf extract identified various beneficial compounds with enormous medicinal importance. Hence, an attempt was made in the present study to exploit these beneficial properties of guava leaf in formulation of herbal tooth paste.

Apart from guava leaf extract, the active ingredient in the toothpaste formulation include *Acacia arabica* gum powder, sea salt, guava leaf powder, stevia herb extract powder and pepper mint oil (Table 1). Three tooth paste formulations F1, F2 and F3 were made by changing the concentration of the ingredients. *Acacia arabica* is one of the species recognized worldwide as a multipurpose tree and been effectively utilized treating cough, diarrhoea, diabetes, dysentery, eczema, skin diseases, wound healing, burning sensation and as an astringent, demulcent, anti-asthmatic (Farag et al. 2015). Especially its gum contains four aldobiouronic acids viz., 6-*o*-(β-glucopyranosyluronicacid)-d-galactose; 6-*o*-(4-*o*-methyl-β-d-glucopyranosyluronicacid)-d-galactose; 4-*o*-(α-d-glucopyranosyluronic acid)-d-galactose; and 4-*o*-(4-*o*-methyl-α-d-glucopyranosyluronic acid)-d-galactose (Rajendran et al. 2010). Gum was included in the formulation as bio-adhesive and binder to mix all the ingredients and makes the preparation intact. Moreover, the presence of aldobiouronic acids in the gum gives cooling effect after using the toothpaste. On the other hand, stevia was used as an alternative for synthetic or powerful sweetener, which indeed reduces the growth of oral bacteria and species specific odour. It has been proved to contain sweet diterpene and acylated glycoside (Karp et al. 2017) which gives sweetening property. This natural sweetener has the ability to maintain glucose level in diabetic’s patients and scientifically proven to be nontoxic (Karp et al. 2017).

The physical and chemical characteristics of different toothpaste formulations such as pH, abrasiveness, foaming ability, spreadability and cleaning ability were studied. pH of the three tooth paste formulations is in alkaline range. pH value of toothpaste plays a crucial role in evaluating its properties as it gives an indication of the constituents. It was reported that ideal toothpaste should always have a pH between 5.5 and 10.5 (Price et al. 2000) which was exactly found in F2 and F3. Das et al. (2013) reported the stimulation of bacterial growth in mouth due to lower pH that leads to dental carries. Thus, an alkaline pH helps in neutralizing acid biofilm, kill germs and reduce unpleasant odours (Bouassida et al. 2017). Besides, guava leaf powder added in the formulation provides sufficient abrasiveness for maximum cleaning with minimum wear on enamel surface.

Another desirable characteristic preferred by the consumers is foam formation as it facilitates the toothpaste to spread all over the oral cavity during mechanical brushing. So, to attract more consumers sodium lauryl/laureth sulfate (SLS) is used as a surfactant to produce foam. But SLS is found to have degenerative effect on the cell membranes because of its protein denaturing properties and carcinogenic nature. Even though, there is no significant correlation found so far but either way usage of chemicals in long run can definitely affect the consumer’s health. Considering all these factors in our formulation we have not used any surfactant, because of which the current formulations has not produced much foam and thus, categorized as non-foaming toothpaste.

As a matter of fact, stain removing ability has a significant role than foaming property. Teeth get exposed to various confectionery products, beverages, food colours, tobacco products etc., which will strongly attach and create stains. Moreover, the demand for products that enhance whitening of the teeth has increased significantly. The current formulation appear to be effective in removing stains from the teeth and improve the whiteness as the guava powder present in the toothpaste acts as an abrasive. Guava extract not only improves the abrasiveness of the formulated toothpaste but also enhances the microbial properties.

The tooth paste formulations were evaluated for their antibacterial activity against *Bacillus subtilis, Proteus vulgaris, Staphylococcus aureus, Streptococcus mutants* and *Streptococcus oralis* and found that the formulation F3 as very effective in comparison to the other formulations. Nisha et al. (2011) reported that essential oil of *P. guajava* is efficient in inhibiting the growth of both Gram-positive and Gram-negative bacteria at higher concentration. Oluwasina et al. (2019) studied the effect toothpaste formulated from extracts of *Syzygium aromaticum*, *Jatropha curcas latex* and *Dennettia tripetala* against *E. coli*, *Bacillus* sp. *S. aureus*, *S. Epidermidis* etc. Bora et al. (2014) reported that the key factor to choose dentifrice is its antibacterial efficacy, as opportunistic microorganisms will proliferate and produce a harsh environment which leads to the destruction of enamel. The formulated toothpaste showed clear inhibition zone against all the test bacteria, which indicated the antimicrobial activity of the toothpaste.

Cytotoxicity of the tooth paste formulations have been tested and confirmed that the formulations have no significant effect on the reduction of Vero cell viability (*P *< 0.05) up to the concentration of 320 μg/mL. Moreover, the tooth paste formulation (F3) was found to be stable up to 12 months without change in its physico-chemical properties. These finding authenticate F3 as an efficient tooth paste formulation than the others.

As an outcome of the present work, a polyherbal toothpaste was prepared with guava leaf powder possessing antimicrobial and antioxidant properties as a major ingredient. Other ingredients used in the tooth paste formulation were successful in removing stains from the teeth, cleansing oral cavity and acting as carriers of various therapeutic compounds. The uniqueness of the present study lies in formulating the toothpaste with natural herbs, absolutely void of chemicals in contrary to many commercially available tooth pastes which are made of chemicals. These chemicals act as potential carcinogens. Moreover, the tooth paste formulation (F3) was found to exhibit stability, abrasiveness with minimum effect on the enamel and negligible toxicity substantiating the suitability of the formulation for human application.

## Data Availability

The data related to current study are available from the corresponding author on reasonable request.
